# Correlation between parameters of self-monitoring of blood glucose and the perception of health-related quality of life in patients with type 1 diabetes mellitus

**DOI:** 10.1590/2359-3997000000222

**Published:** 2016-11-07

**Authors:** Juliana Santos Paula, Letícia Dinis Braga, Rodrigo Oliveira Moreira, Rosane Kupfer

**Affiliations:** 1 Instituto Estadual de Diabetes e Endocrinologia Luiz Capriglione Rio de Janeiro RJ Brasil Serviço de Diabetes, Instituto Estadual de Diabetes e Endocrinologia Luiz Capriglione (IEDE), Rio de Janeiro, RJ, Brasil

**Keywords:** Type 1 diabetes mellitus, hypoglycemia, self-monitoring of blood glucose, quality of life

## Abstract

**Objective:**

The aim of this study was to evaluate how different parameters of short-term glycemic control would correlate with the perception of health-related quality of life (HRQoL) in patients with type 1 diabetes mellitus (T1D).

**Subjects and methods:**

A total of 50 T1D patients aged 18 to 50 years were evaluated with the questionnaires Problem Areas in Diabetes (PAID) scale and Diabetes Quality of Life (DQOL) measure after 30 days of self-monitoring of blood glucose (SMBG). Glycemic control was evaluated using glycated hemoglobin (HbA_1c_), mean glucose levels (MGL) in the prior month’s data from SMBG (Accu-Check 360^o^), number of hypoglycemic episodes (< 70 mg/dL and < 50 mg/dL), and glycemic variability (GV).

**Results:**

PAID correlated positively with MGL (r = 0.52; p < 0.001) and HbA_1c_ (r = 0.36; p < 0.0097), but not with GV (r = 0.17; p = 0.23) or number of hypoglycemic episodes (r = 0.15; p = 0.17 for glucose < 70 mg/dL and r = 0.02; p = 0.85 for glucose < 50 mg/dL). After multiple linear regression, only MGL remained independently related to PAID scores. DQOL scores had a positive correlation with MGL (r = 0.45; p = 0.001), but not with HbA_1c_ (r = 0.23; p = 0.09), GV (r = 0.20; p = 0.16), or number of hypoglycemic episodes (r = 0.06 p = 0.68).

**Conclusion:**

In T1D patients, MGL, but not HbA_1c_ or number hypoglycemic episodes, was the glycemic control parameter that best correlated with short-term perception of HRQoL.

## INTRODUCTION

Type 1 diabetes mellitus (T1D) is a chronic disease with high rates of morbidity and mortality. It comprises around 5 to 10% of all cases of diabetes mellitus (DM) (1), and according to estimates from the International Diabetes Federation, 86,000 new patients are diagnosed with T1D every year (2). From the moment of diagnosis, managing DM places a huge burden on the patient, with serious limitations on and changes in lifestyle (3). Treatment consists of multiple injections of insulin, self-monitoring of glucose levels, balanced diet, and regular physical activity. Moreover, patients with DM live in fear of future complications, risk of hypoglycemia, and premature death.

Health-related quality of life (HRQoL) is an important health indicator. It is a subjective and multidimensional concept that encompasses a set of interrelated factors, including physical, functional, psychological, and religious aspects (4). Evaluation of HRQoL in patients with chronic diseases and its important relationship with therapeutic management are becoming more valued and recognized as a significant area of scientific knowledge (5). The importance of HRQoL in patients with chronic diseases like DM is such that it has not only been considered a significant predictor of health outcomes, it is now considered a significant health outcome itself (6,7).

It is very interesting to speculate how different aspects related to T1D and its treatment would influence the perception of HRQoL in these patients. On one side, recurrent episodes of hypoglycemia and the fear of these episodes have already been demonstrated to impair HRQoL (8). On the other side, hyperglycemia and the fear or microvascular complications have also been related to a worse HRQoL (9,10). However, it is not clear which of these aspects of T1D treatment would more significantly impact the HRQoL of these patients.

Our study’s objective was to evaluate how different parameters of short-term glycemic control would correlate with the perception of HRQoL in patients with T1D. The main hypothesis of our study was that the perception of a good glycemic control through self-monitoring of blood glucose (SMBG) might be more important than HbA_1c_ measurement and/or hypoglycemic episodes in a sample of patients with low socioeconomic status. In this population, the perception of good daily glucose control may be more easily interpreted than biochemical parameters of glycemic control (*i.e.*, HbA_1c_).

## SUBJECTS AND METHODS

### Study population

This was a prospective study involving 50 consecutive outpatient subjects with T1D, aged between 15 and 50 years, performed at the *Instituto Estadual de Diabetes e Endocrinologia* (IEDE), Brazil, between May and October 2013. Patients were included if they had more than 1 year of diagnosis and were under treatment with a basal-bolus insulin regimen (multiple insulin injections).

Patients with type 2 DM (T2D), with serious or limiting complications from DM, end-stage kidney disease, chronic hepatic insufficiency, depression or anxiety, and pregnant women were excluded. Patients who did not have sufficient understanding to fill out the questionnaires or who refused to sign the Informed Consent Form (ICF) were also excluded.

The Ethics Committee approved the study’s protocol. All patients read, understood, and signed the ICF form before undergoing clinical evaluation.

### Evaluation of glycemic control and quality of life

After the initial evaluation, the patients were instructed to perform for the following 30 days at least five measurements of glucose levels a day, before the main meals and when symptoms of hypoglycemia were present. Accu-Check Active was used for the measurements, and data were analyzed using the Accu-Check 360^o^ software. The following parameters were evaluated: mean glucose levels (MGL) and standard deviation (SD), and glycemic variation based on the coefficient of variation (CV) and the number of episodes of hypoglycemia < 70 mg/dL and < 50 mg/dL. Glycemic variability (GV) was also calculated with the formula for the CV = (100 X SD)/mean glucose.

After 30 days, glycemic control was evaluated with glycated hemoglobin (HbA_1c_), which was measured using the high-performance liquid chromatography method. In addition, the participants filled out both questionnaires described below.

The Problem Areas in Diabetes (PAID) scale (11) is a specific measurement of psychosocial adjustment to DM. It consists of 20 items covering common problematic situations and negative emotional aspects commonly experienced by these patients. The authors of this questionnaire designed it to evaluate the subject’s emotional understanding of DM. Scores range from 0 to 100.

The Diabetes Quality of Life (DQOL) measure (12) consists of 44 items and contains subscales covering five different areas: satisfaction with the treatment, impact, worries about future diabetes complications, worries about the social and vocational aspects of the disease, and general well-being. The total result of the responses for each item of the subscales was calculated. In both questionnaires, a higher result indicates a lower HRQoL.

### Statistical analysis

The statistical analysis was performed with GraphPad InStat 3.00 (GraphPad Software, San Diego, CA, USA). The Kolmogorov-Smirnov test was used to determine parametric and nonparametric variables. According to the test, age, MGL, and HbA_1c_ levels were considered nonparametric. Spearman test was used for correlation analysis of nonparametric variables and Pearson test for parametric variables. Multiple linear regression was used to identify independent variables of glycemic control that could be related to HRQoL. The level of statistical significance was 5%.

## RESULTS

Of the 50 patients evaluated, 25 were male. The mean age of the sample was 36.8 ± 11.3 years. No significant differences were observed between genders, and the mean monthly family income (in number of minimum wages) was 2.4 ± 2.3. The patients performed a mean of 131.4 ± 28.4 blood glucose tests during the 30-day period. The HbA_1c_ levels of the sample ranged from 5.8 to 12.9%, with a median of 7.5% (mean = 7.6 ± 1.4%), while MGL ranged from 108.1 to 274.4 mg/dL, with a median of 145.8 mg/dL. The mean CV of the sample was 49.7 ± 8.0. Of the 50 patients, 49 had at least one hypoglycemic episode (glucose levels < 70 mg/dL). The mean number of hypoglycemic episodes was 16.5 ± 10.3, ranging from 1 to 38 episodes.

**Figure 1:**
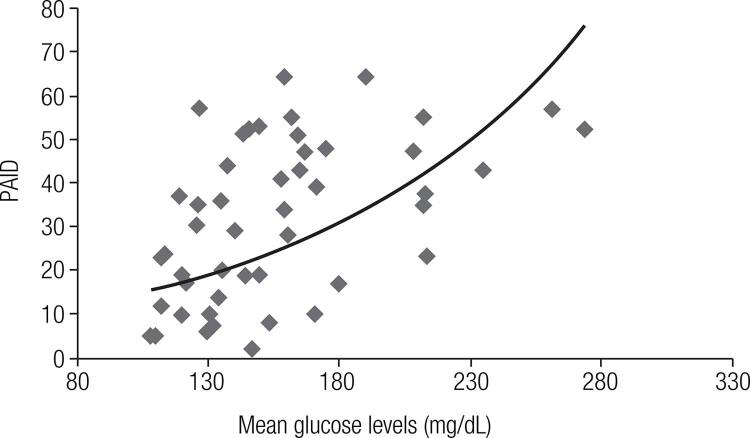
Correlation between Problem Areas in Diabetes (PAID) scores and mean glucose levels (mg/dL). Increased PAID scores indicate a worse perception of quality of life (r = 0.52; p < 0.0001).

Correlation analysis was used to correlate glycemic control parameters and PAID scores. PAID scores correlated positively with MGL (r = 0.52; p < 0.001; Figure 1) and HbA_1c_ levels (r = 0.36; p < 0.0097), but not with GV (r = 0.17; p = 0.23) or the number of hypoglycemic episodes (r = 0.15; p = 0.17 for glucose < 70 mg/dL and r = 0.02; p = 0.85 for glucose < 50 mg/dL). After multiple linear regression (using MGL and HbA_1c_ as independent variables), only MGL remained independently related to PAID (t ratio = 2.769; p = 0.008).

**Figure 2:**
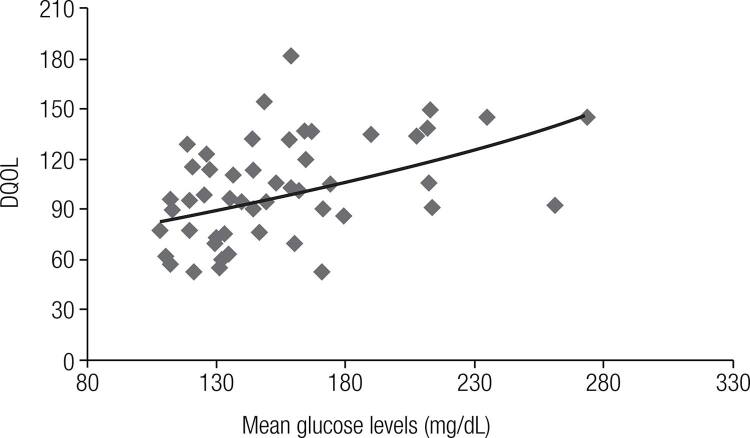
Correlation between the values for the Diabetes Quality of Life (DQOL) measure and mean glucose levels (mg/dL). Increased DQOL scores indicate a worse perception of quality of life (r = 0.45; p = 0.001).

Similarly, we also evaluated the correlation between the parameters for glycemic control and the DQOL. We found a statistically significant positive correlation between DQOL scores and MGL (r = 0.45; p = 0.001; Figure 2), but not with HbA_1c _levels (r = 0.23; p = 0.09), CV (r = 0.20; p = 0.16) or number of episodes of hypoglycemia < 70 mg/dL (r = 0.06 p = 0.68) and < 50 mg/dL (r = 0.08; p = 0.57).

## DISCUSSION

The diagnosis of DM is associated with several modifications in the patient’s daily life. The fear of diabetes complications, as well as the fear of hypoglycemia, may be related to a significant worsening of the HRQoL. We aimed at investigating which parameter of glycemic control was associated with a worse perception of HRQoL. Our main finding was that MGL, but not HbA_1c_ levels or number of hypoglycemic episodes, was the only indicator independently associated with a worse perception of HRQoL.

Different studies have already described an association between higher levels of HbA_1c_ and a substantially lower HRQoL (13-17), with higher scores on the PAID (18). Our study did not find an independent correlation between HRQoL and HbA_1c_ levels. One hypothesis to explain this difference could be that, in populations of low socioeconomic status, it may not be so simple for patients to understand the concept of HbA_1c_. Other studies have explored the relationship between HbA_1c_ and specific mediators of HRQoL in diabetes, and as with our findings, they found such a relationship to be inconsistent or nonexistent (18,19).

In our study, higher scores on the DQOL and PAID correlated with high blood glucose levels. Another Brazilian study yielded similar results, showing evidence of a direct relationship between higher glycemic levels in patients who were more dissatisfied with T1D (20). One possible explanation would be that the patients felt uncomfortable when visualizing high glucose levels since hyperglycemia is well known to be associated with complications of the disease. It seems that MGL are more easily interpreted than HbA_1c_ levels as an indicator of glycemic control. We estimated that the main isolated predictor of a reduction in HRQoL in these patients was the appearance of complications arising from long-term hyperglycemia (21,22). We should stress that, by using the term “predictor” to describe any aspect related to HRQoL, we are considering it as an outcome. However, the causal relationship between these variables remains unclear. HRQoL can affect a patient’s perception of the disease, its management, behavior related to self-care, metabolic control and the incidence of complications; all these variables, in turn, can affect the HRQoL (23,24). One can also speculate that hyperglycemia is directly associated with severe symptoms. Although this seems a plausible hypothesis, the vast majority of the patients in this study (42 individuals) had MGL below 200 mg/dL, which are usually asymptomatic. Finally, another hypothesis, albeit unlikely, may also help to explain our results. Patients with a worse HRQoL may be less adherent to treatment, which would lead to a worse glycemic control. Prospective and interventional studies are necessary to clarify the causality of this relationship.

Two important studies have already been published investigating the HRQoL in a large sample of Brazilian patients with T1D. The first study evaluated a sample of patients younger than those in our study and demonstrated that a better glycemic control could positively impact the health status of individuals with T1D. Interestingly, glycemic control was evaluated using HbA_1c_ levels. Another important finding in this study was that economic status was inversely related to health status (7). A secondary analysis using the same population demonstrated differences in health status in different Brazilian regions; these differences, however, were not explained by HbA_1c_ levels. The authors suggested that additional factors not evaluated in their study could determine the HRQoL of patients with T1D (25). It is worth noticing that SMBG was not evaluated in these studies.

Hypoglycemic episodes are another widely studied variable. The fear of these episodes leads to a lack of adherence to the proposed treatment, in an effort to avoid a recurrence, with a consequent compromise in HRQoL (15,26). An Australian study is in line with the results of our research, finding no evidence of a relationship between hypoglycemic episodes and HRQoL (27). It seems reasonable to speculate that, at least in some populations, the occurrence of hypoglycemia may indicate a better perception of glycemic control.

Our study has some limitations. First, only a small number of individuals were evaluated. Furthermore, these individuals were evaluated after only 1 month of SMBG. It seems reasonable to speculate that different results would have been achieved with larger populations and longer follow-up. These two important limitations (*i.e.*, sample size and study length) may also have impacted the relationship between hypoglycemia and HRQoL. Previous studies have already demonstrated that hypoglycemia may have a negative impact in the HRQoL (8). It should be pointed out that we did not differentiate symptomatic from asymptomatic episodes, which could be an important factor linking glycemic control and HRQoL.

In conclusion, the relationship between HRQoL and parameters of glucose monitoring in patients with T1D is complex. Our study demonstrates a positive correlation between hyperglycemia and a poorer perception of HRQoL in a sample of Brazilian patients with T1D. Interestingly, it seems that MGL had the most significant correlation with the perception of HRQoL, suggesting that daily glucose testing, and not HbA_1c _measurement, might be used to investigate the impact of different treatment and interventions in the patient’s perception of the disease. Our results suggest that, when treating a patient with T1D, physicians should optimize glycemic control not only to prevent diabetic complications, but also to improve the patient’s HRQoL. Moreover, more attention should be given to HRQoL in order to optimize glycemic control. Finally, it also indicates that SMBG may be an important tool in patients’ education and may be a simple way to demonstrate the efficacy of the treatment to the patients.
